# Bayesian variable selection for high dimensional predictors and self-reported outcomes

**DOI:** 10.1186/s12911-020-01223-w

**Published:** 2020-09-07

**Authors:** Xiangdong Gu, Mahlet G Tadesse, Andrea S Foulkes, Yunsheng Ma, Raji Balasubramanian

**Affiliations:** 1 Department of Biostatistics and Epidemiology, University of Massachusetts, Amherst, MA USA; 2 Department of Mathematics and Statistics, Georgetown University, Washington, DC USA; 3 Biostatistics Center, Division of Clinical Research, Massachusetts General Hospital Research Institute, Boston, MA USA; 4 Department of Medicine, University of Massachusetts Medical School, Worcester, MA USA

**Keywords:** Bayesian variable selection, Self-reports, High dimensional data

## Abstract

**Background:**

The onset of silent diseases such as type 2 diabetes is often registered through self-report in large prospective cohorts. Self-reported outcomes are cost-effective; however, they are subject to error. Diagnosis of silent events may also occur through the use of imperfect laboratory-based diagnostic tests. In this paper, we describe an approach for variable selection in high dimensional datasets for settings in which the outcome is observed with error.

**Methods:**

We adapt the spike and slab Bayesian Variable Selection approach in the context of error-prone, self-reported outcomes. The performance of the proposed approach is studied through simulation studies. An illustrative application is included using data from the Women’s Health Initiative SNP Health Association Resource, which includes extensive genotypic (>900,000 SNPs) and phenotypic data on 9,873 African American and Hispanic American women.

**Results:**

Simulation studies show improved sensitivity of our proposed method when compared to a naive approach that ignores error in the self-reported outcomes. Application of the proposed method resulted in discovery of several single nucleotide polymorphisms (SNPs) that are associated with risk of type 2 diabetes in a dataset of 9,873 African American and Hispanic participants in the Women’s Health Initiative. There was little overlap among the top ranking SNPs associated with type 2 diabetes risk between the racial groups, adding support to previous observations in the literature of disease associated genetic loci that are often not generalizable across race/ethnicity populations. The adapted Bayesian variable selection algorithm is implemented in R. The source code for the simulations are available in the Supplement.

**Conclusions:**

Variable selection accuracy is reduced when the outcome is ascertained by error-prone self-reports. For this setting, our proposed algorithm has improved variable selection performance when compared to approaches that neglect to account for the error-prone nature of self-reports.

## Background

The time to a silent event in several clinical settings can only be assessed through sequentially administered diagnostic tests. For example, diabetes can be detected by measuring levels of fasting blood glucose or glycosylated hemoglobin levels (HbA1c). Although gold standard diagnostic tests are often available, the associated cost is prohibitive in large epidemiological studies which often recruit hundreds of thousands participants. Instead, disease incidence is often ascertained through less expensive but error-prone procedures such as self-report. One example is the Women’s Health Initiative (WHI), which enrolled 161,808 postmenopausal women aged 50-79 years at 40 clinical centers across the U.S. from 1993-1998 with ongoing follow-up [1]. Due to cost considerations, prevalent and incident type 2 diabetes is ascertained by self-reported questionnaires at each annual visit. In this paper, we propose and apply a Bayesian variable selection (BVS) approach for variable selection in high dimensional datasets while simultaneously accounting for the error-prone nature of self-reported outcomes. We apply the proposed methods to discover single nucleotide polymorphisms (SNPs) associated with type 2 diabetes risk in the WHI Clinical Trial and Observational Study SNP Health Association Resource (SHARe), which includes extensive genotypic (>900,000 SNPs) and phenotypic data on 9,873 African American and Hispanic American women.The proposed methods equally apply when a silent outcome is ascertained through laboratory-based diagnostic procedures that are subject to misclassification.

When a time-to-event outcome is ascertained by a perfect diagnostic test that is administered at pre-specified time points during the course of follow up, the outcome is interval-censored. In this context, methods to estimate the survival distribution and assess covariate effects have been developed [2, 3]. However, when an error-prone diagnostic procedure such as a self-report is used instead, standard methods for interval censored outcomes lead to bias. Previous work in this area include methods for modeling error-prone outcomes with application to studies in HIV, HPV and STD [4–7]. A formal likelihood framework was developed to estimate the distribution of the time-to-event of interest in the presence of error-prone laboratory-based diagnostic tests, in the context of pediatric HIV clinical trials [5]. Also in the context of pediatric HIV studies, the discrete proportional hazard model was extended to incorporate mis-measured outcomes and also covariates [7]. In related work, generalized Cox models were considered in settings involving time-to-event outcomes with incomplete event adjudication [8–10]. Other related work includes that proposed in the context of HPV studies [6], where the authors accommodate misclassification by incorporating ideas of binary generalized linear models with outcomes subject to misclassification [11]. A formal likelihood framework was proposed to accommodate sequentially administered, error-prone self-reports or laboratory based diagnostic tests for modeling the association of a targeted set of covariates with the time-to-event outcome of interest [12]. While a rich literature exists to handle estimation and hypothesis testing in the presence of error-prone survival outcomes, none of these approaches can be applied directly to variable selection in high-dimensional data, in which the number of features (*p*) far exceeds the number of subjects (*n*). In this setting, standard likelihood based estimation approaches are intractable.

The BVS method has been previously proposed for variable selection in high dimensional datasets [13, 14]. The Bayesian model proceeds by assigning a mixture prior distribution to the regression coefficients (***β***) corresponding to the high dimensional predictors, for example - a mixture of a point mass at 0 and a uniform distribution [13], a mixture of two normal distributions centered at 0 but with distinct variances [14]. The estimated posterior probabilities of the latent binary indicators for inclusion in the model is used for variable selection. Several papers have applied this approach for discovering a sparse feature set associated with an outcome in high-dimensional microarray data for various settings. Previous works include models for binary outcomes [15], multi-category responses [16], and censored outcomes [17]. Notably, the use of the BVS method in large-scale settings such as genome-wide association studies (GWAS) was successfully demonstrated [18]. The BVS approach has also been extended to in application to clustered data to simultaneously discover group structure and identify discriminating variables [19–21]. One advantage of the BVS approach when compared to other variable selection methods is that it can be naturally extended to incorporate external information such as biological pathway membership [22, 23]. A comprehensive review of BVS algorithms can be found in the literature [24]. Improvements to and novel applications of the BVS procedure continues to be an active area of research [25–28].

In this paper, we incorporate a BVS approach into a likelihood-based model proposed by Gu, X. et al. (2015). This allows us to conduct variable selection in high dimensional data while accounting for the imperfect observation of a time-to-event outcome. Through simulation studies, we illustrate the impact of ignoring error in the outcome on variable selection and compare the performance of the BVS spike-and-slab prior with that of our proposed algorithm. We apply the BVS approach to discover SNPs associated with incident type 2 diabetes in a dataset of 9,873 African American and Hispanic American women enrolled in the WHI.

The organization of this paper is as follows: In the “Methods” section, we present notation and the form of the likelihood function that accommodates error in self-reported outcomes. We incorporate this likelihood into the BVS algorithm, to handle high-dimensional datasets. We conduct simulation studies to compare the variable selection performance of different approaches for high dimensional datasets arising from GWAS and metabolomic studies. We apply our proposed methods for the discovery of single nucleotide polymorphisms (SNPs) associated with type 2 diabetes risk in the WHI Clinical Trial and Observational Study SNP Health Association Resource (SHARe), among African American and Hispanic American women. Lastly, we discuss the findings of this study and highlight future directions.

## Methods

In this section, we introduce notation, present the form of the likelihood function to accommodate error-prone, self-reported outcomes that has been previously described [12] and integrate with a BVS approach for variable selection.

### Notation, likelihood function

Let *X* refer to the random variable denoting the unobserved time-to-event for an individual, with associated survival, density and hazard functions denoted by *S*(*x*),*f*(*x*) and *λ*(*x*), for *x*≥0. The time origin is set to 0, corresponding to the baseline visit at which all subjects enrolled in the study are assumed to be event-free. This implies that Pr(*X*>0)=1.

Without loss of generality, we set *X*=*∞* when the event of interest does not occur. Let N denote the number of subjects and *n*_*i*_ denote the number of visits for the *i*^*th*^ subject during the follow-up period. At each visit, we assume that a subject would self-report their disease status as either positive or negative. For example, at each semi-annual (WHI-CT) or annual contact (WHI-OS), all participants were asked, “Since the date given on the front of this form, has a doctor prescribed any of the following pills or treatments?” Choices included “pills for diabetes” and “insulin shots for diabetes”. Thus, incident treated diabetes was ascertained, and was defined as a self-report of a new physician diagnosis of diabetes treated with oral drugs or insulin.

Let *τ*_1_,⋯,*τ*_*J*_ denote the distinct, ordered visit times in the dataset among *N* subjects, where 0=*τ*_0_<*τ*_1_<...<*τ*_*J*_<*τ*_*J*+1_=*∞*. Thus, the time axis can be divided into *J*+1 disjoint intervals, [0,*τ*_1_),[*τ*_1_,*τ*_2_),⋯,[*τ*_*J*_,*∞*). Let ***Z*** denote the *P*×1 vector of covariates with corresponding *P*×1 vector of regression coefficients denoted by ***β***. To incorporate the effect of covariates, we assume the proportional hazards (PH) model, $\lambda \left (x|\boldsymbol {Z}=\boldsymbol {z}\right)=\lambda _{0}(x)e^{\boldsymbol {z}'\boldsymbol {\beta }}\phantom {\dot {i}\!}$, or equivalently, $S\left (x|\boldsymbol {Z}=\boldsymbol {z}\right)=S_{0}(x)^{e^{\boldsymbol {z}'\boldsymbol {\beta }}}$. Thus, the log-likelihood function for a random sample of *N* subjects can be expressed as:

1 $$  l\left(\boldsymbol{\theta},\boldsymbol{\beta}\right)=\sum_{i=1}^{N}\log\left(\sum_{j=1}^{J+1}D_{ij}\left(\sum_{k=j}^{j+1}{\theta_{k}}\right)^{e^{\boldsymbol{z}_{i}'\boldsymbol{\beta}}}\right),  $$

where *θ*_*j*_=Pr(*τ*_*j*−1_<*X*≤*τ*_*j*_) and where $\sum _{j=1}^{J+1} \theta _{j} = 1$. The elements *D*_*ij*_ of the matrix *D* are functions of the observed data including the visit times, the corresponding self-reported results, and the constants *φ*_0_,*φ*_1_ that correspond to the specificity and sensitivity of self-reports, respectively. The details of the derivation were originally reported in a previous publication [12] and have been included in Section 1 of the Supplement.

When *P*<<*N* and assuming that *φ*_0_,*φ*_1_ are known, the maximum likelihood estimates of the unknown parameters *β*_1_,⋯,*β*_*P*_,*θ*_1_,⋯,*θ*_*J*_ can be obtained by numerical maximization of the log-likelihood function in Eq. (1), subject to the constraints that $\sum _{j=1}^{J+1}{\theta _{j}}=1$ and *θ*_*j*_>0. Statistical inference regarding the parameters of interest (*β*_1_,⋯,*β*_*P*_,*θ*_1_,⋯,*θ*_*J*+1_) can be made by using asymptotic properties of the maximum likelihood estimators [29]. For settings in which *P*>*N*, a Bayesian approach incorporating a spike and slab variable selection procedure is described below.

### Bayesian variable selection (BVS)

In this section, we adapt the spike and slab BVS approach in the context of error-prone, self-reported outcomes. We introduce a latent vector ***γ***=(*γ*_*p*_,1≤*p*≤*P*), where each *γ*_*p*_ is an indicator variable denoting whether the *p*^*th*^ covariate is included (*γ*_*p*_=1) or not (*γ*_*p*_=0) in the model. The BVS analysis proceeds via MCMC methods to estimate the posterior distribution of ***γ***. With this latent variable formulation for variable selection, the log likelihood function in Eq. (1) is a function of the parameters *θ*_1_,⋯,*θ*_*J*_,***β***,***γ*** and is denoted *l*(***θ***,***β***,***γ***). We assume the following hierarchical structure of the prior distributions corresponding to the unknown parameters in the model:

$$\begin{array}{l} \boldsymbol{\theta} \sim \text{Dirichlet}(\boldsymbol{1}) \\ \beta_{p} \mid \gamma_{p} \sim \gamma_{p} N(0, b^{2}) + (1-\gamma_{p})\delta_{0} \\ \gamma_{p} \mid \omega \sim \text{Bernoulli}(\omega) \\ \omega \sim \text{Beta}(w_{1}, w_{2}) \end{array} $$

where *δ*_0_ is the Dirac function corresponding to a point mass at 0 and where *b*,*w*_1_,*w*_2_ are treated as known hyper-parameters.

By treating the interval probabilities ***θ*** as nuisance parameters, we propose the following Metropolis-Hasting algorithm. At iteration *t*, we let the indices *t*−1 and ^∗^ denote the current and proposed values of the parameters, respectively.

1. Initialization: Set *ω*^(0)^ to a randomly generated value from Beta(*w*_1_,*w*_2_) distribution. Set ***γ***^(0)^=***0*** and ***β***^(0)^=***0***. Optimize the log-likelihood function in Eq. (1) with respect to ***θ*** by fixing ***β***=***β***^(0)^, and then set ***θ***^(0)^ to equal the optimized value for ***θ***.
2. Update selected variable and associated regression coefficient: Select a covariate *p*∈(1,⋯,*P*) at random and let the proposed value $\gamma _{p}^{*}=1-\gamma _{p}^{t-1}$.
  - If $\gamma _{p}^{*}=0$, the corresponding regression coefficient $\beta _{p}^{*}$ is set to 0. If $\gamma _{p}^{*}=1$, the proposed regression coefficient $\beta _{p}^{*}$ is sampled from *N*(0,*b*^2^) distribution.
  - Optimize the log-likelihood function in Eq. (1) with respect to ***θ*** by fixing ***β***=***β***^∗^, denote the optimized value to be ***θ***^∗^. The optimized value ***θ***^∗^ is used as the proposed value for ***θ***.
  - Accept the proposed values $\left (\gamma _{p}^{*}, \beta _{p}^{*}, \theta ^{*}\right)$ with probability min(*e*^*Δ*^,1) where:
    $$\begin{array}{@{}rcl@{}} \Delta &=& l\left(\boldsymbol \theta^{*}, \boldsymbol \beta^{*}\right) - l\left(\boldsymbol \theta^{(t-1)}, \boldsymbol \beta^{(t-1)}\right) \\ &&+ \left(2\gamma_{p}^{*}-1\right)\log\left(\frac{\omega^{(t-1)}}{1-\omega^{(t-1)}} \right) \end{array} $$
    See Section 2 of the Supplement for details regarding the derivation of *Δ*.
3. Update regression coefficients and interval probabilities: For each included covariate, update the coefficient with a user defined probability *p*_*main*_, for example 0.30. If a main effect *β*_*p*_ is chosen for update,
  - Let the proposed value, $\beta _{p}^{*}$, be a random sample from the distribution $N\left (\beta _{p}^{(t-1)}, b^{2}\right)$.
  - Optimize the log-likelihood function in Eq. (1) with respect to ***θ*** by fixing ***β***=***β***^∗^, where we denote the optimized value to be ***θ***^∗^. The optimized value ***θ***^∗^ is used as the proposed value for ***θ***.
  - Accept the proposed values $\left (\beta _{p}^{*}, \boldsymbol \theta ^{*}\right)$ with probability min(*e*^*Δ*^,1) where
    $$\begin{array}{@{}rcl@{}} \Delta &=& l\left(\boldsymbol \theta^{*}, \boldsymbol \beta^{*}\right) - l\left(\boldsymbol \theta^{(t-1)}, \boldsymbol \beta^{(t-1)}\right) \\ &&+ \frac{\left(\beta_{p}^{(t-1)}\right)^{2} - \left(\beta_{p}^{*}\right)^{2} }{2b^{2}} \end{array} $$
    See Section 2 of the Supplement for details regarding the derivation of *Δ*.
4. Update *ω*: Using Gibbs sampling, update *ω* by generating a sample from Beta (*w*_1_+*K*_*γ*_,*w*_2_+*P*−*K*_*γ*_), where *K*_*γ*_ is the number of main effects selected.

After the burn-in period, the covariates are ranked from most to least important by their inclusion probabilities based on the posterior distribution of *γ*_*p*_.

## Results

### Simulation studies

We report results from simulation studies to evaluate the performance of the proposed BVS algorithm in the presence of outcomes subject to error, under various parameter settings. First, we consider a high dimensional dataset in which each feature is a random variable with three levels, reflecting the two possible homozygous (AA, aa) and the single heterozygous (Aa) combination of alleles of a SNP. In Section 3.2 of the Supplement, we consider a high dimensional dataset in which each feature is a continuous random variable, scaled to have mean 0 and unit variance.

The results presented here are averages of 1,000 simulated datasets, where each dataset included *n*=100 subjects and *P*=100 covariates. To mimic real data settings, the 100×100 design matrix was obtained by random sampling of a subset of 100 covariates for 100 participants from the WHI Clinical Trial and Observational Study SHARe (described below) and a metabolomics study of cardiovascular disease (see Section 3.2. of the Supplement). The design matrix was standardized before simulation.

Results from error-prone self-reports for each subject were simulated assuming four pre-scheduled visit times per subject over 8 years of follow up, with no missed visits. The distribution of true event times in the reference group (i.e. ***Z***=***0***) was assumed to be exponential with baseline hazard denoted by *λ*_0_. The value of *λ*_0_ was determined by fixing the corresponding cumulative incidence rate (CIR) in the reference group to equal *CIR*=10*%* or30*%*, over the 8-year study duration. The true event time for each subject in the study was simulated from an exponential distribution, where the hazard function (*λ*) was determined through the PH model (*λ*=*λ*_0_*e*^***βZ***^). In each dataset, five out of 100 covariates were randomly sampled as true associations with the outcome, with a corresponding coefficient *β*=1.0 (or, hazard ratio of *e*^*β*^≈2.7) in the PH model. The regression coefficients for the remaining 95 covariates in the PH model were set to 0. For each subject, an error-prone self-report (positive or negative) at each visit was simulated from a Bernoulli distribution. At each visit, the probability of a positive self-report was governed by the sensitivity (*φ*_1_) if the visit time is after the true event time or the complement of the specificity (*φ*_0_) if the visit time precedes the true event time. The values of (*φ*_1_,*φ*_0_) were varied between [(1,1),(1,0.9),(0.75,1),(0.61,0.995)]. We note that the sensitivity and specificity values (0.61,0.995) correspond to the properties of diabetes self-reports in the WHI [30].

For each parameter setting, we compared the variable selection performance of the following three strategies: (1) Random survival forests assuming no error in self-report [31]; (2) the proposed BVS algorithm assuming no error in the self-report (*φ*_1_=*φ*_0_=1); and (3) the proposed BVS accounting for the imperfect nature of self-reports. Our rationale for selecting these algorithms for comparison include evaluating the: (1) performance of the proposed BVS approach when compared to a distinct, yet multivariable, non-parametric, tree-based ensemble approach such as the Random Forests; and (2) potential increase in variable selection accuracy when accounting for error in self-reports through the proposed BVS approach.

1. **Random survival forests (RSF)**: The algorithm implemented in the *randomForestSRC* R package [32] was applied to each dataset by defining the time-to-event outcome as the time from baseline (origin) to the time of the first positive self-report (observed event) or the time of last observation (censored observation). In particular, this results in no difference in the handling of the two study designs (“No missed visits” versus “NTFP”). The Random survival forests was implemented using the function *rfsrc* in the package with default parameter setting, e.g. there are 1,000 trees in the forest. The 100 covariates were ranked by the variable importance metric output by the algorithm. The computing time for a single 100×100 dataset on a Macbook Pro 2017 is about 2 seconds.
2. **Proposed BVS assuming no error in self-reports (BVS**_***perfect***_**)**: This analysis was based on the proposed BVS algorithm by setting *φ*_1_=*φ*_0_=1. Note that in this case, we only consider self-reports up to the first positive report since negative self-reports that follow a positive self-report have zero probability. The MCMC algorithm was run up to 100,000 iterations and the first 20,000 iterations were discarded as burn-in. We set the values of the hyper-parameters governing the prior distributions to the following: *b*=1.0,*w*_1_=5,*w*_2_=100. The 100 covariates were ranked based on the posterior distribution of ***γ***. The computing time for a single 100×100 dataset on a Macbook Pro 2017 is about 3 minutes.
3. **Proposed BVS algorithm (BVS**_***e***_**)**: This analysis was based on the proposed BVS algorithm by setting *φ*_1_,*φ*_0_ to equal the values assumed in the data generation process. This algorithm is implemented in two ways: (1) Self-reports collected at all pre-scheduled visits are included in the analysis (referred to as “NMISS”); (2) Self-reports following the first positive are discarded (referred to as “No Test after First Positive or NTFP”). The MCMC algorithm was run up to 100,000 iterations and the first 20,000 iterations were discarded as burn-in. We set the values of the hyperparameters governing the prior distributions to the following: *b*=1.0,*w*_1_=5,*w*_2_=100. The computing time for a single 100×100 dataset on a Macbook Pro 2017 is about 3 minutes.
  The R code for implementing the simulations described in this Section has been included in Section 4 of the Supplement. Model diagnostics of the convergence of the MCMC chain and run length control indicated that the choice of 100,000 iterations with a burn-in of 20,000 was justified (See Section 3.1 in the Supplement).

The 100×100 design matrix for this simulation study was randomly selected from the existing GWAS data from the WHI Clinical Trial and Observational Study SHARe, which includes extensive genotypic (>900K SNPs) and phenotypic data for 12,007 African American and Hispanic American women. All missing genotypes were imputed to be homozygous for the major allele “AA”. Among the 100 SNPs selected in the design matrix for the simulation, 74 had minor allele frequency (MAF) between 0 and 0.35 and the remaining 26 SNPs had MAF between 0.35 and 0.5. Each SNP was incorporated into the PH model as a numeric covariate that was generated by coding genotype “AA” as 0, genotype “Aa” as 1, and “aa” as 2. In this model, the homozygous major allele (AA) category serves as the reference group and the homozygous ‘aa’ category has twice the effect on outcome as the heterozygous ‘Aa’ category [33]. We note that this implies the assumption of a linear effect across the ordered genotype categories. While relaxing this assumption is straightforward from a modeling perspective, it would result in a significant increase in computational complexity.

Figure 1 in the Supplement presents the posterior distribution of ***γ*** for the 100 SNPs from a single representative simulation. Here, the data generating mechanism for the time-to-event outcome and self-reports was based on the first five SNPs, each with corresponding regression coefficient *β*=0.7 in the PH model. The results were based on *φ*_1_=0.61,*φ*_0_=0.995, a 30% rate of cumulative incidence in the reference group, and assuming that there were no missed visits. We observed that true associations (SNPs 1-5) had significantly higher posterior probabilities of inclusion when compared to the *average* corresponding value for those SNPs that were not associated with outcome.

Table 1 shows the proportion of simulated datasets in which a SNP that is associated with the outcome was found as ranking among the top five SNPs by the posterior probability of inclusion - results are averaged over the five SNPs with true associations with the outcome. In all settings with the exception of one (CIR=0.1,*φ*_1_=1,*φ*_0_=0.9), the BVS and BVS_*e*_ algorithms perform better than RSF. When self-reports after the first positive are excluded from analysis (NTFP), both BVS and BVS_*e*_ have comparable performance indicating that the performance loss due to assuming incorrect sensitivity and specificity values is negligible when test results are discarded. However, when self-reports at all visit times are included in analysis (“NMISS”), and when specificity (*φ*_0_) is less than perfect, BVS_*e*_ results in a significantly higher probability of discovering true associations when compared to BVS. For example, when CIR =0.1, *φ*_1_=1.00, *φ*_0_=0.90, the probability of a SNP ranking among the top five by BVS_*e*_ under NMISS is 0.81 (±0.01) as compared to 0.34 (±0.01) and 0.41 (±0.01) by BVS (assuming perfect self-reports) and RSF, respectively. The false positive rates were comparable across algorithms for each simulation setting.

**Table 1:** Probability of ranking among the top five SNPs by posterior probability of inclusion, for SNPs that are associated with outcome. CIR denotes the cumulative incidence rate in the reference group, RSF denotes Random Survival Forests, BVS_*perfect*_ denotes the proposed BVS algorithm assuming perfect self-reports and BVS_*e*_ denotes the proposed BVS procedure. NTFP denotes a study design setting in which all self-reports following the first positive result are discarded and NMISS denotes the setting where there are no missed visits

CIR	sensitivity (*φ*_1_)	specificity (*φ*_0_)	RSF	BVS_*perfect*_	BVS_*e*_	BVS_*e*_
					NTFP	NMISS
0.1	1	1	0.70(±0.01)	0.87(±0.01)	0.87(±0.01)	0.87(±0.01)
0.1	1	0.9	0.41(±0.01)	0.34(±0.01)	0.30(±0.01)	0.81(±0.01)
0.1	0.75	1	0.68(±0.01)	0.81(±0.01)	0.84(±0.01)	0.84(±0.01)
0.1	0.61	0.995	0.63(±0.01)	0.69(±0.01)	0.74(±0.01)	0.75(±0.01)
0.3	1	1	0.80(±0.01)	0.98(±0.01)	0.98(±0.01)	0.98(±0.01)
0.3	1	0.9	0.58(±0.01)	0.63(±0.01)	0.68(±0.01)	0.97(±0.01)
0.3	0.75	1	0.78(±0.01)	0.90(±0.01)	0.95(±0.01)	0.95(±0.01)
0.3	0.61	0.995	0.74(±0.01)	0.82(±0.01)	0.88(±0.01)	0.88(±0.01)

Similar results were observed for the setting where the features were continuous measurements, representing data observed in metabolomic studies (Section 3.2 of the Supplement).

### Application

The proposed methods were applied to data from the WHI Clinical Trial and Observational Study SHARe, to identify SNPs associated with risk of incident type 2 diabetes mellitus. The dataset includes extensive genotypic (909,622 SNPs) and phenotypic information on 12,008 African American and Hispanic American women. After excluding participants who self-reported diabetes at baseline, the analysis was restricted to 9,873 participants.

Prevalent diabetes at baseline and incident diabetes were assessed through self-reported questionnaires in the WHI. At baseline and at each annual visit, every participant was asked whether she had ever received a physician diagnosis of and/or treatment for diabetes when not pregnant since the time of the last self-report/visit. Using data from a WHI sub-study [30], estimates of sensitivity, specificity, and baseline negative predictive value of self-reported diabetes outcomes were obtained by comparing self-reported outcomes to fasting glucose levels and medication data. A participant was considered to be truly diabetic if she had either taken anti-diabetic medication and/or had a fasting glucose level ≥ 126mg/dL. By using a subset of 5485 participants, with information at baseline on diabetes self-reports, fasting glucose levels and medication inventory, we estimated that self-reports have a sensitivity (*φ*_1_) of 0.61 and a specificity (*φ*_0_) of 0.995 [30]. These parameter values are used in our analysis.

After excluding participants with self-reported diabetes at baseline, the remaining subsets of 6,704 African American and 3,169 Hispanic American women were analyzed independently. The results presented here are based on follow up until 2013. The average follow up from baseline was 11.6 years, with a maximum follow up of 16 years - during this period, 21.2% of the African American and 18.5% of the Hispanic American women self-reported a new diagnosis of diabetes.

The data pre-processing procedures resulted in 300,000 SNPs being included in the statistical analysis (Section 5 of the Supplement, Supplementary Figs. 2-3). Results from the proposed BVS algorithm (BVS_*e*_) are compared to the BVS algorithm assuming perfect self-reports (BVS_*perfect*_) and to two SNP-by-SNP approaches, including a model based on the likelihood in Eq. (1) (*icensmis*) [34] and the Cox proportional hazards (PH) model. Details regarding data pre-processing and the statistical models are presented in Section 5 of the Supplement.

Figures 4-5 in the Supplement show bar plots of the posterior probability of inclusion from the proposed algorithm (BVS_*e*_) for the 300,000 SNPs included in each analysis in the African American and Hispanic American datasets, respectively. Since the dimensionality reduction procedure was carried out within each dataset, not all SNPs entered the BVS_*e*_ analysis in both datasets. SNPs that were found to rank among the top 10 most important by at least one of the aforementioned analyses in the datasets of African American women and Hispanic American women are shown in Tables 2 and 3, respectively. Each SNP is annotated with its host gene (if known) and its upstream and downstream genes. In both populations, the rankings by the proposed BVS_*e*_ algorithm differed significantly from the rankings by BVS assuming perfect self-reports and from each of the univariate (SNP by SNP) analyses (Tables 2-3), underscoring the value in utilizing a broad set of analytical approaches for variable selection in high dimensional datasets. The BVS algorithm assuming perfect self-reports identified only 2 SNPs in the African American dataset and one SNP in the Hispanic American dataset, with non-zero posterior probability of inclusion. Interestingly, none of the SNPs discovered among the top 10 (by at least one approach) in the African American dataset overlapped with the corresponding set in the Hispanic American subcohort.

**Table 2:** Rankings of individual SNPs in the WHI Clinical Trial and Observational Study SHARe among African American women in the WHI (*n*=6704). Results from the following analyses are reported: (1) The proposed BVS approach (BVS_*e*_); (2) BVS assuming perfect tests (BVS_*perfect*_); (3) univariate (SNP by SNP) analysis assuming a Cox PH model; and (4) univariate analysis adjusting for error in self-report (*icensmis*). Each analysis simultaneously adjusted for the top two principal components to account for population stratification. SNPs are ordered from most (rank= 1) to least important (rank >1000) with regard to their association with time to incident type 2 diabetes. Ranks >1000 are denoted by −

BVS_*e*_	BVS_*perfect*_	Cox PH	*icensmis*	rs	Intron	Upstream	Downstream
Rank	Rank	Rank	Rank	Number			
1	2	1	1	rs2805434	RYR2		
2	-	15	10	rs5946729			SHOX, CRLF2
3	-	28	20	rs10126793		PDK3	SUPT20HL1
4	-	3	3	rs10054129		RXFP3, ADAMTS12	
5	-	22	16	rs7523871		RNU5F-1	LOC101929689, LYPLAL1
6	-	-	-	rs6795523	IGSF11		
7	-	-	-	rs149091		ANKRD55	LOC102467147
8	-	81	69	rs10820848		LOC101927847	UNQ6494
9	-	-	-	rs10950835		SP4	RPL23P8
10	-	-	-	rs2714365		CHST9	CDH2
34	-	9	9	rs17693218		LYZL1	C10orf126
-	-	2	2	rs2805429	RYR2		
-	-	5	5	rs7737188		RXFP3, ADAMTS12	
-	-	8	7	rs16917265		INIP, SNX30	
-	-	6	6	rs4144636	ASTN2		
-	-	7	8	rs12247963	FAM188A		
-	-	4	4	rs15958			
-	-	10	17	rs1959083			LINC00520, RPL13AP3
-	1	13	19	rs6573059			LINC00520, RPL13AP3

**Table 3:** Rankings of individual SNPs in the WHI Clinical Trial and Observational Study SHARe among Hispanic American women in the WHI (*n*=3169). Results from the following analyses are reported: (1) The proposed BVS approach (BVS_*e*_); (2) BVS assuming perfect tests (BVS_*perfect*_); (3) univariate (SNP by SNP) analysis assuming a Cox PH model; and (4) univariate analysis adjusting for error in self-report (*icensmis*). Each analysis simultaneously adjusted for the top two principal components to account for population stratification. SNPs are ordered from most (rank= 1) to least important (rank >1000) with regard to their association with time to incident type 2 diabetes. Ranks >1000 are denoted by −

BVS_*e*_	BVS_*perfect*_	Cox PH	*icensmis*	rs	Intron	Upstream	Downstream
Rank	Rank	Rank	Rank	Number			
1	-	4	2	rs6547248	CTNNA2		
2	-	22	28	rs488672	TRIM29	TRIM29, OAF	
3	-	19	24	rs1396128		LINC00968	IMPAD1
4	-	-	-	rs2964611			GLRA1, G3BP1
5	-	-	-	rs519206		LYZL1	C10orf126
6	-	3	7	rs6079637	MACROD2		
7	-	-	-	rs4778193	OCA2		
8	-	-	-	rs1972897	PARD3		
9	-	-	-	rs10202023	LOC101927196		
10	-	9	8	rs6899814		RNY4	SH3BGRL2, C6orf7
13	-	14	10	rs17253815	GPC6		
29	-	5	1	rs17175231	GPC6		
86	-	1	3	rs6135332	MACROD2		
-	-	6	6	rs276637	SHISA9		
-	-	2	4	rs10242930		TMEM106B, THSD7A	
-	-	7	5	rs10809502		TYRP1	PTPRD-AS2
-	-	10	9	rs1561955		TYRP1	PTPRD-AS2
-	-	8	12	rs6079638	MACROD2		
-	1	-	-	rs9610221		HMGXB4	ISX

In the dataset of African American women, a total of 19 SNPs were identified in the top 10 by at least one of the four strategies, while simultaneously adjusting for population stratification (Table 2). SNP rs2805434 was ranked within the top 2 ranks by all four analysis approaches. The host gene RYR2 has been implicated in insulin secretion in previous studies [35]. RYR2 is host to another SNP (rs2805429) identified as second most important by both univariate approaches in this population. rs2805434 was removed from the analysis dataset due to the pre-processing procedures in the Hispanic American women.

Another finding with support in the literature is that of SNP rs10126793 - its upstream gene PDK3 or pyruvate dehydrogenase kinase 3 is in the class of PDK isoenzymes that have been shown to be strong therapeutic targets for preventing and treating metabolic diseases [36]. rs10126793 had a weak association with type 2 diabetes risk in the Hispanic American dataset with a posterior probability of inclusion of 0 in both BVS analyses and *p*-values of 0.07 and 0.02 in univariate *icensmis* and Cox PH analyses, respectively.

In the dataset of Hispanic American women, a total of 19 SNPs were identified in the top 10 by at least one of the three strategies, while simultaneously adjusting for population stratification (Table 3). SNP rs6547248 was ranked as the top candidate by the BVS_*e*_ algorithm and among the top five SNPs by Cox PH and *icensmis*. The host gene CTNNA2 (or Catenin Alpha 2) is a protein coding gene and may function as a linker between cadherin adhesion receptors and the cytoskeleton to regulate cell-cell adhesion and differentiation in the nervous system [37]. The host gene CTNNA2 was found among 24 novel candidate genes associated with type 2 diabetes risk in a African American subsample of 973 participants with type 2 diabetes and 104 healthy control participants in the GENNID study [38].

SNP rs488672 within host gene TRIM29 was ranked as second most important by BVS_*e*_ and among the top 30 SNPs by Cox PH and *icensmis*. RNA sequence data from an animal study involving a mouse model of type 2 diabetes showed that TRIM29 acts as an E3 ligase that targets both insulin receptor (IR) and insulin receptor substrate 1 (IRS1) for ubiquitin-dependent degradation, resulting in insulin resistance [39]. In this study, the authors showed that TRIM29 levels are ≥2.5 fold higher in the kidney cortex of diabetic mice when compared to wild type mice, respectively.

SNPs rs6079637 was found among the top 10 SNPs by all models with the exception of BVS_*perfect*_. Similarly, SNP rs6135332 was found among the top 100 SNPs by BVS_*e*_ and among the top five SNPs by Cox PH and *icensmis*. Both SNPs are located in the intron of gene MACROD2 and are significantly correlated (*p*<0.0001), with *R*^2^=0.936 and $\phantom {\dot {i}\!}D^{'} = 1.0$ [40]. In a study of 1,100 Han Chinese individuals from 398 families in the Stanford Asian Pacific Program for Hypertension and Insulin Resistance study, genetic loci within the MACROD2 gene were associated with vascular adhesion protein-1 levels (VAP-1) in females. VAP-1 is a membrane-bound amine oxidase highly expressed in mature adipocytes and released into the circulation. VAP-1 has been strongly implicated in several pathological processes, including diabetes, inflammation, hypertension, hepatic steatosis and renal diseases, and is an important disease marker and therapeutic target [41].

SNP rs6899814 was ranked in the top 10 by each strategy with the exception of BVS_*perfect*_, flanked by upstream gene RNY4 and downstream genes SH3BGRL2 and C6orf7. Of note, SH3BGRL2 was identified as a gene that is implicated in type 1 diabetes, type 2 diabetes and gestational diabetes in a transcriptome meta-analysis of peripheral lymphomononuclear cells [42]. rs6899814 was an insignificant predictor of incident type 2 diabetes in the African American dataset, with a posterior probability of inclusion of 0 in both BVS analyses and *p*-values of 0.34 and 0.16 in univariate *icensmis* and Cox PH analyses, respectively.

In our independent analyses of African American and Hispanic American participants in the WHI, we identified several novel SNPs associated with type 2 diabetes risk. An overlap in the findings in the two datasets were two SNPs in the same genomic region that are flanked upstream by gene LYZL1 and downstream by C10orf126 - these SNPs were rs17693218 and rs519206 in the datasets of African American and Hispanic American participants, respectively. Among the other top ranking SNPs, there was little overlap between the race/ethnicity groups (Tables 2-3). These results add to previous observations in the literature of disease associated genetic loci that are often not all generalizable across race/ethnicity populations [43].

## Discussion

In this paper, we propose a BVS procedure for variable selection in high dimensional datasets, in settings where a time-to-event outcome is observed with error. The models developed in this paper are motivated by self-reported outcomes of incident type 2 diabetes collected in the Women’s Health Initiative, that are subject to misclassification. The proposed methods apply to other settings in which the event of interest is diagnosed using an imperfect laboratory-based diagnostic test that is administered at prescheduled times during follow-up.

We presented results from simulations, considering different data types (GWAS, metabolomics) and a variety of settings with regard to cumulative incidence of event during the study and sensitivity/specificity of the self-report (or imperfect diagnostic test). When silent outcomes are ascertained through imperfect self-reports with imperfect specificity, our proposed algorithm has a significantly better performance with regard to variable selection when compared to the approaches that assume no error in the outcome ascertainment. In studies where collection of self-reports or diagnostic test results ceases after the first positive result, our modified algorithm no longer performs better than other approaches that ignore the error in outcomes. We applied the proposed algorithm to data from the WHI Clinical Trial and Observational Study SHARe in separate analyses of the data from African American and Hispanic American women. We found a distinct genetic signature associated with type 2 diabetes risk among the African American and Hispanic American populations in the WHI, with little overlap in risk alleles between groups.

The computational burden of BVS approaches can be considerable. In future work, it would be useful to explore efficient alternatives to the stochastic search algorithms - for example, the expectation maximization variable selection approach [26] could result in significant improvement in computational efficiency. Other useful extensions of the BVS procedure could involve incorporating known biological relationships between predictors, as discussed in previous work such as [22, 23, 25].

## Conclusion

In high dimensional data applications, variable selection can be negatively impacted when the outcome of interest is observed with error such as in self-reports. In settings where the specificity of self-reported outcomes is less than perfect, a significant degradation in variable selection accuracy was observed. For this setting, our proposed algorithm employs a Bayesian variable selection approach that incorporates a likelihood function that models the error prone nature of the self-reported outcomes. The proposed algorithm had significantly better variable selection performance when compared to similar algorithms that ignore the error in the outcome. The proposed algorithm was applied to GWAS data in the WHI Clinical Trial and Observational Study SHARe to discover novel SNPs associated with risk of incident type 2 diabetes in African American and Hispanic American populations.

## Supplementary information

**Additional file 1** Supplement to Bayesian variable selection for high dimensional predictors and self-reported outcomes

**Additional file 2** The online supplementary material includes the following: **File name**: bmc_article_supplement.pdf**Description:** Supplementary methods and results

## Data Availability

The Women’s Health Initiative - SNP Health Association Resource (WHI-SHARe) data (dbGaP Study Accession: phs000200.v12.p3) can be obtained by requesting access through the dbGaP website https://dbgap.ncbi.nlm.nih.gov/.

## Abbreviations

SNP: Single Nucleotide Polymorphism
HbA1c: glycosylated hemoglobin
WHI: Women’s Health Initiative
BVS: Bayesian Variable Selection
SHARe: SNP Health Association Resource
HIV: Human Immunodeficiency Virus
HPV: Human Papilloma Virus
STD: Sexually Transmitted Disease
GWAS: Genome wide association study
WHI-CT: Women’s Health Initiative Clinical Trials
WHI-OS: Women’s Health Initiative Observational Study
MCMC: Markov Chain Monte Carlo
CIR: Cumulative Incidence Rate
PH: Proportional Hazards
RSF: Random Survival Forests
NTFP: No Tests following First Positive
NMISS: No Missing tests
MAF: Minor Allele Frequency
CTNNA2: Catenin Alpha 2
VAP-1: Vascular Adhesion Protein 1
