# Supplementary material for: Bayesian variable selection for high dimensional predictors and self-reported outcomes
Source: BMC Med Inform Decis Mak. 2020 Sep 7;20:212. doi: 10.1186/s12911-020-01223-w (PMC7487595; doi:10.1186/s12911-020-01223-w)
Supplement: Supplementary file 2 — Additional file 2 The online supplementary material includes the following: File name: bmc_article_supplement.pdfDescription: Supplementary methods and results [file 12911_2020_1223_MOESM2_ESM.pdf]

RESEARCH

Supplement to Bayesian variable selection for high dimensional predictors and self-reported outcomes

Xiangdong Gu[1], Mahlet G Tadesse[2], Andrea S Foulkes[3], Yunsheng Ma[4] and Raji Balasubramanian[1*]

*Correspondence: rbalasub@umass.edu
[1]Department of Biostatistics and Epidemiology, University of Massachusetts, Amherst, MA, USA
Full list of author information is available at the end of the article

Abstract

Section 1 of the supplement presents details regarding the derivation of the form of the observed data likelihood incorporating error in self-reports (Gu, X et al., 2015). Section 2 presents the derivation of the acceptance probabilities in Steps 2 and 3 of the MH algorithm. Section 3.1 presents convergence diagnostics of the MCMC chains for selected simulation settings. Section 3.2 presents the simulation results for the setting of a high dimensional dataset in which each feature is a continuous random variable, scaled to have mean 0 and unit variance. These simulations are motivated by data observed in metabolomic studies. The R code for the simulation studies reported in the paper is provided in Section 4. Section 5 includes details regarding the application to data from the Women's Health Initiative. Figures 1 - 5 are referenced in the main paper.

1 Likelihood incorporating self-reports

Here we present the details involving the assumptions and derivation of the observed data log likelihood that incorporates error-prone self-reports. This derivation has been previously published in [1].

Let X refer to the random variable denoting the unobserved time to event for an individual, with associated survival, density and hazard functions denoted by $S(x), f(x)$ and $\lambda(x)$, for $x \geq 0$. The time origin is set to 0, corresponding to the baseline visit at which all subjects enrolled in the study are assumed to be event-free. In other words, $\Pr(X > 0) = 1$. This assumption reflects the design and goals of population-based studies in which one is interested in estimating predictors of incident disease (i.e. $X > 0$).

Without loss of generality, we set $X = \infty$ when the event of interest does not occur. Let N denote the number of subjects and n_i denote the number of visits for the ith subject during the follow-up period. At each visit, we assume that each subject would self-report his/her disease status as either positive or negative. For example, at each semi-annual (WHI-CT) or annual contact (WHI-OS), all participants were asked, "Since the date given on the front of this form, has a doctor prescribed any of the following pills or treatments?" Choices included "pills for diabetes" and "insulin shots for diabetes". Thus, incident treated diabetes was ascertained, and was defined as a self-report of a new physician diagnosis of diabetes treated with

oral drugs or insulin.

For the ith subject, we let $\mathbf{R_i} = \{R_{i1}, \cdots, R_{in_i}\}$ and $\mathbf{t_i} = \{t_{i1}, \cdots, t_{in_i}\}$ denote the $1 \times n_i$ vectors of self-reported, binary outcomes and corresponding visit times, respectively. R_{ik} is equal to 1 if the kth self-report for the ith subject is positive (indicating occurrence of the event of interest such as diabetes) and 0 otherwise. We assume that self-reports are collected at pre-scheduled visits up to the time of the first positive self-report - thus, the vectors of test results or self-reports ($\mathbf{R_i}$), visit times ($\mathbf{t_i}$) and the number of self-reports collected per subject (n_i) are random. Let $\tau_1, \cdots, \tau_J$ denote the distinct, ordered visit times in the dataset among N subjects, where $0 = \tau_0 < \tau_1 < ... < \tau_J < \tau_{J+1} = \infty$. Thus, the time axis can be divided into $J + 1$ disjoint intervals, $[0, \tau_1), [\tau_1, \tau_2), \cdots, [\tau_J, \infty)$.

The joint probability of the observed data for the ith subject can be expressed as:

$$\begin{aligned} g(\mathbf{R_i}, \mathbf{t_i}, n_i) &= \sum_{j=1}^{J+1} \Pr(\tau_{j-1} < X_i \le \tau_j) \Pr(\mathbf{R_i}, \mathbf{t_i}, n_i \mid \tau_{j-1} < X_i \le \tau_j) \\ &= \sum_{j=1}^{J+1} \theta_j \Pr(\mathbf{R_i}, \mathbf{t_i}, n_i \mid \tau_{j-1} < X_i \le \tau_j) \end{aligned}$$

where $\theta_j = \Pr(\tau_{j-1} < X \le \tau_j)$, $\tau_0 = 0$ and $\tau_{J+1} = \infty$.

To simplify, we make the assumption that an individual's n_i self-reports are independent conditional on the true time to event X_i. That is,

$$\Pr(\mathbf{R_i} \mid X_i, \mathbf{t_i}) = \prod_{k=1}^{n_i} \Pr(R_{ik} \mid X_i, t_{ik})$$

This assumption implies that the observed values of other self-reported outcomes do not provide additional information about the distribution of a particular self-reported outcome from that provided by the actual time of the event. We note that this assumption is similar in spirit to the common assumption in measurement error regression models that the conditional distribution of the response variable, given the covariate and its proxy, is the same as the conditional distribution given only the covariate.

It can be shown that the joint probability of the observed data for the ith subject can be simplified as:

$$\begin{aligned} g(\mathbf{R_i}, \mathbf{t_i}, n_i) &= \sum_{j=1}^{J+1} \theta_j \left[\prod_{k=1}^{n_i} \Pr(R_{ik} \mid \tau_{j-1} < X_i \le \tau_j, t_k) \right] \\ &= \sum_{j=1}^{J+1} \theta_j C_{ij} \end{aligned} \tag{1}$$

where $C_{ij} = [\prod_{k=1}^{n_i} \Pr(R_{ik} \mid \tau_{j-1} < X_i \leq \tau_j, t_k)]$. See details presented in [1]. We assume that the probability of a positive self-report at the kth visit ($R_{ik} = 1$) conditional on the interval containing the true event time and visit time can be expressed as:

$$\Pr(R_{ik} = 1|\tau_{j-1} < X_i \leq \tau_j, t_k) = \begin{cases} \varphi_1, & t_k \geq \tau_j \\ 1 - \varphi_0, & t_k \leq \tau_{j-1} \end{cases}$$

Here, φ_1 and φ_0 denote the sensitivity and specificity of self-reports, respectively. Thus the terms C_{ij}, for $j = 1, \cdots, J+1$ in equation (1) can be expressed as a product involving the constants φ_1 and φ_0. Thus, in the absence of covariates, the log likelihood for a random sample of N subjects can be expressed as:

$$l(\boldsymbol{\theta}) = \log(L(\boldsymbol{\theta})) \quad = \quad \sum_{i=1}^{N} \log(\sum_{j=1}^{J+1} C_{ij}\theta_j)$$

For the special case where self-reports are perfect ($\varphi_1 = \varphi_0 = 1$), the likelihood above reduces to the non-parametric likelihood for interval censored observations given in Turnbull (1976).

Let $\boldsymbol{Z}$ denote the $P \times 1$ vector of covariates with corresponding $P \times 1$ vector of regression coefficients denoted by $\boldsymbol{\beta}$. To incorporate the effect of covariates, we assume the proportional hazards model, $\lambda(x|\boldsymbol{Z} = \boldsymbol{z}) = \lambda_0(x)e^{\boldsymbol{z}'\boldsymbol{\beta}}$, or equivalently, $S(x|\boldsymbol{Z} = \boldsymbol{z}) = S_0(x)^{e^{\boldsymbol{z}'\boldsymbol{\beta}}}$.

To derive the form of the log-likelihood based on the assumption of the proportional hazards model, we re-parameterize the log likelihood in (1) in terms of the survival function, $\boldsymbol{S} = (1 = S_1, S_2, \cdots, S_{J+1})^T$, where $S_j = \Pr(X > \tau_{j-1})$. Since $S_j = \sum_{l=j}^{J+1} \theta_l$, the vector of interval probabilities can be expressed as $\boldsymbol{\theta} = T_r\boldsymbol{S}$, where T_r is the $(J+1) \times (J+1)$ transformation matrix. Let $C = [C_{ij}]$ denote the $N \times (J+1)$ matrix of the coefficients, C_{ij}, and let the $N \times (J+1)$ matrix D be defined as $D_{N\times(J+1)} = C \times T_r$. Then, the log-likelihood function for the one-sample setting can be expressed as:

$$l(\mathbf{S}) = \sum_{i=1}^{N} \log(\sum_{j=1}^{J+1} D_{ij}S_j), \tag{2}$$

where $S_1 = 1$ and $S_2, S_3, \cdots, S_{J+1}$ are the unknown parameters of interest.

Let $1 = S_1 > S_2 > ... > S_{J+1}$ denote the baseline survival functions (i.e. corresponding to $\boldsymbol{Z} = \boldsymbol{0}$), evaluated at the left boundaries of the intervals $[0, \tau_1), [\tau_1, \tau_2), \cdots, [\tau_J, \infty)$. Then, for subject i, with corresponding covariate vector $\boldsymbol{z}_i$, $S_j^{(i)} = (S_j)^{e^{\boldsymbol{z}_i'\boldsymbol{\beta}}}$. Thus, the log-likelihood function for a random sample of N subjects in equation (2) can be extended to incorporate covariates as

$$l(\boldsymbol{S}, \boldsymbol{\beta}) = \sum_{i=1}^{N} \log(\sum_{j=1}^{J+1} D_{ij}(S_j)^{e^{\boldsymbol{z}_i'\boldsymbol{\beta}}}) \quad = \sum_{i=1}^{N} \log(\sum_{j=1}^{J+1} D_{ij}(1 - \sum_{k=1}^{j} \theta_k)^{e^{\boldsymbol{z}_i'\boldsymbol{\beta}}}). \tag{3}$$

The elements of the D matrix are functions of the observed data including the visit times, the corresponding self-reported results ($\mathbf{t_i}$, $\mathbf{R_i}$ for $i = 1, \cdots, n_i$), and the constants φ_0, φ_1.

2 Metropolis Hastings algorithm

In both steps 2 and 3 of the MH algorithm, the acceptance probabilities (e^{Δ}) are obtained according to the following:

$$\begin{aligned} e^{\Delta} &= \frac{p(\theta^* \mid x, y)}{p(\theta^{(t-1)} \mid x, y)} \frac{Q(\theta^* \to \theta^{(t-1)})}{Q(\theta^{(t-1)} \to \theta^*)} \\ &= \frac{\mathcal{L}(\theta^*) p(\theta^*)}{\mathcal{L}(\theta^*) p(\theta^{(t-1)})} \frac{Q(\theta^* \to \theta^{(t-1)})}{Q(\theta^{(t-1)} \to \theta^*)} \end{aligned}$$

, where $Q(\cdot)$ denotes the transition probability, θ^* denotes the proposed value and $\theta^{(t-1)}$ denotes the current value of θ.

In Step 2 of the MH algorithm, the randomly selected predictor and its corresponding regression coefficient are updated. Note that If $\gamma_j^* = 1$, then $\beta_j^* \sim N(0, b^2)$, $\gamma_j^{(t-1)} = 0$ and $\beta_j^{(t-1)} = 0$. Thus,

$$\begin{aligned} e^{\Delta} &= \frac{\mathcal{L}(\theta^*)}{\mathcal{L}(\theta^{(t)})} \times \frac{p(\beta_j^* \mid \gamma_j^* = 1) p(\gamma_j^* = 1)}{p(\beta_j^{(t)} = 0 \mid \gamma_j^{(t)} = 0) p(\gamma_j^{(t)} = 0)} \times \frac{1/p \times 1 \times 1}{1/p \times 1 \times p(\beta_j^* \mid \gamma_j^* = 1)} \\ &= \frac{\mathcal{L}(\theta^*)}{\mathcal{L}(\theta^{(t)})} \times \frac{\omega^{(t)}}{1 - \omega^{(t)}} \end{aligned}$$

On the other hand, if $\gamma_j^* = 0$, then $\beta_j^* = 0$, $\gamma_j^{(t)} = 1$ and $\beta_j^{(t)} \sim N(0, b^2)$. In this case,

$$\begin{aligned} e^{\Delta} &= \frac{\mathcal{L}(\theta^*)}{\mathcal{L}(\theta^{(t)})} \times \frac{p(\beta_j^* = 0 \mid \gamma_j^* = 0) p(\gamma_j^* = 0)}{p(\beta_j^{(t)} \mid \gamma_j^{(t)} = 1) p(\gamma_j^{(t)} = 1)} \times \frac{1/p \times 1 \times p(\beta_j^{(t)} \mid \gamma_j^{(t)} = 1)}{1/p \times 1 \times 1} \\ &= \frac{\mathcal{L}(\theta^*)}{\mathcal{L}(\theta^{(t)})} \times \frac{1 - \omega^{(t)}}{\omega^{(t)}} \end{aligned}$$

In Step 3 of the MH algorithm, the regression coefficients for all included covariates and interval probabilities are updated. The acceptance probability in Step 3c) is e^{Δ}, obtained as follows:

$$\begin{aligned} e^{\Delta} &= \frac{\mathcal{L}(\theta^*)}{\mathcal{L}(\theta^{(t)})} \times \frac{p(\beta_j^* \mid \gamma_j^* = 1)}{p(\beta_j^{(t)} \mid \gamma_j^{(t)} = 1)} \times \frac{p(\beta_j^{(t)} \mid \beta_j^*)}{p(\beta_j^* \mid \beta_j^{(t)})} \\ &= \frac{\mathcal{L}(\theta^*)}{\mathcal{L}(\theta^{(t)})} \times \frac{e^{-\beta_j^{*2}}/2b^2}{e^{-\beta_j^{(t)2}}/2b^2} \times \frac{e^{-(\beta_j^{(t)} - \beta_j^*)^2/2b^2}}{e^{-(\beta_j^* - \beta_j^{(t)})^2/2b^2}} \\ &= \frac{\mathcal{L}(\theta^*)}{\mathcal{L}(\theta^{(t)})} \times e^{-\frac{1}{2b^2}(\beta_j^{*2} - \beta_j^{(t)2})} \end{aligned}$$

3 Simulation

3.1 Model diagnostics

The simulation studies reported in the Results section were based on individual MCMC chains that were run up to 100,000 iterations, where the first 20,000 iterations were discarded as burn-in. To justify the choice of the number of iterations and burn-in, we carried out several diagnostic tests.

The Heidelberg and Welch convergence diagnostic [3] was used to test the null hypothesis that the sampled values of ω are drawn from a stationary distribution. The total number of iterations needed and the estimated burn-in were calculated using the Raftery and Lewis diagnostic [4] - this run length control diagnostic estimates the number of iterations required to estimate the 2.5^{th} percentile to within an accuracy of $+/-0.005$, with probability 0.95. Model diagnostics were estimated using the *R* package *coda*.

Table 1 presents model diagnostics for the simulation settings considered in the paper, exploring various values of cumulative incidence, sensitivity and specificity. The Heidelberg and Welch convergence diagnostic p values were all greater than 0.05, indicating no evidence to reject the null hypothesis of a stationary distribution - however, for three of the eight settings considered the p values were < 0.10. For two of these settings, the estimated number of iterations and burn-in were under 100,000 and 20,000, respectively.

Additionally, trace plots of the log posterior and of the number of included variables in the model were examined.

3.2 Simulation study: continuous features motivated by metabolomics

The 100×100 design matrix for the simulation study was randomly selected from data profiled in a cardiovascular disease metabolomics study that was conducted to discover prognostic biomarkers in blood plasma for near-term cardiovascular events [2]. Participants were selected from the CATHGEN project, which collected peripheral blood samples from consenting research participants undergoing cardiac catheterization at Duke University Medical Center from 2001 through 2011. 68 cases were selected from among individuals who had a major adverse cardiac event (MACE) within two years following the time of their sample collection. In a 1:1 matched study design, 68 controls were selected from individuals who were MACE-free for the two years following sample collection and were matched to cases on age, gender, race/ethnicity and severity of coronary artery disease. High-content mass spectrometry and multiplexed immunoassay-based techniques were employed to quantify 472 metabolites from each subject's serum specimen. Comprehensive metabolite profiling of the individual samples was based on a combination of four platforms employing mass spectrometry (MS) based techniques to profile lipids, fatty acids, amino acids, sugars and other metabolites. A detailed description of the mass spectrometry based platforms can be found in a previous publication [2]. Metabolite levels in the 100×100 randomly selected design matrix were natural

logarithm transformed and standardized to render their distributions with mean zero and unit variance.

Table 2 shows the proportion of simulated datasets in which a metabolite that is associated with the outcome was found as ranking among the top five metabolites by the posterior probability of inclusion - results are averaged over the five metabolites with true associations with the outcome. Similar trends to that observed in Section 3.1 were observed here. In particular, the largest gains in performance by the proposed approach (BVS_e) when compared to BVS (assuming perfect self-reports) and RSF were observed when there are no missed visits, and when specificity (φ_0) is less than perfect - for example, when CIR= 0.3, $\varphi_1 = 1.00$, $\varphi_0 = 0.90$, the probability of a metabolite ranking among the top five by BVS_e when all self-reports are included in analysis (NMISS) is 0.84 ($\pm$0.01) as compared to 0.57 ($\pm$0.01) and 0.44 ($\pm$0.01) by BVS (assuming perfect self-reports) and RSF, respectively. The false positive rates were comparable across algorithms for each simulation setting.

4 Simulation R Code

We first need to install the *icensmis* package from github (require *devtools* package):
`devtools::install_github("xiangdonggu/icensmis")`

In order to make the code easier to organize, we put them into 3 files - **utils.R** for defining the functions to perform simulations for different settings, **config.yaml** for specifying simulation parameters, **sim.R** for performing the simulation.

utils.R:

```
#' Function to load yaml simulation setting parameters
#' @param yaml_file yaml file
#'
load_params <- function(yaml_file) {
  require(yaml)
  params <- yaml.load_file(yaml_file)
  for (name in names(params)) {
    assign(name, params[[name]], envir = globalenv())
  }
}


#' Function to simulate outcome data based on give X matrix
#'
#' @param Xmat X matrix, a numeric matrix
#' @param noeven 1 - cumulative incidence
#' @param testtimes visit times
#' @param sensitivity the sensitifity
#' @param specificity the specificity
#' @param betas the betas of biomarkers
#' @param design MCAR or NTFP
#' @examples
#' betas <- c(rep(1, 5), rep(0, 95))
#' data <- simdata(Xmat, .9, 1:4, .61, .995, betas)
#'
simdata <- function(Xmat, noevent, testtimes, sensitivity,
                    specificity, betas, design = "NTFP") {
  N <- nrow(Xmat)
  nfeature <- ncol(Xmat)
  if (nfeature != length(betas))
    stop("Inconsistent beta length and number of features")
  blambda <- -log(noevent)/max(testtimes)
  testtimes <- sort(unique(testtimes))
  ntest <- length(testtimes)
  ID <- rep(1:N, each = ntest)
  time <- rep(testtimes, times = N)
  lambda <- blambda * exp(c(Xmat %*% betas))
  ET <- rexp(N, lambda)
  ET <- ET[ID]
```

```
  occur <- time > ET
  probs <- ifelse(occur, sensitivity, 1 - specificity)
  result <- rbinom(length(occur), 1, probs)
  data <- data.frame(ID, time, result)
  ## NTFP
  if (design == "NTFP") {
    afterpos <- function(x) {
      npos <- cumsum(x == 1)
      (npos == 0) | (npos == 1 & x == 1)
    }
    keep <- unlist(tapply(data$result, data$ID, afterpos))
    data <- data[keep, ]
  }
  row.names(data) <- NULL
  data
}

#' Find first positive time, assuming ascending order of time
#' @param time time
#' @param result result
#' @return first positive time and the status
#'
first_positive <- function(time, result) {
  if (!any(result == 1)) return(c(max(time), 0))
  id <- min(which(result == 1))
  return(c(time[id], 1))
}

#' Find first positive time and status for a data
#' @param data data
#' @param Xmat the design matrix
#' @return first positive time and status
#'
getTS <- function(data) {
  require(dplyr)
  data %>%
    arrange(ID, time) %>%
    group_by(ID) %>%
    summarise(time = first_positive(time, result)[1],
              status = first_positive(time, result)[2])
}


#' Function to obtain RSF fitting of the data
#' @param data the simulated data
#' @param Xmat the design matrix
```

```
#' @return a vector for variable importance
#'
rsf_fit <- function(data, Xmat) {
  require(randomForestSRC)
  data <- getTS(data)
  time <- data$time
  status <- data$status
  modeldata <- data.frame(time, status, Xmat)
  fit <- rfsrc(Surv(time, status) ~., data = modeldata)
  as.vector(vimp(fit)$importance)
}

#' Function to obtain univariance fitting of the data
#' @param data the simulated data
#' @param Xmat the design matrix
unv_fit <- function(data, Xmat) {
  require(survival)
  data <- getTS(data)
  time <- data$time
  status <- data$status
  apply(Xmat, 2, function(x) coxph(Surv(time, status)~x)$wald.test)
}

#' Function to obtain BVS fitting of the data
#' @return a vector of mean gamma for each feature
#' @examples
#' bvs_fit(data, Xmat, .61, .995, b, om1, om2, niter, 1, .1)
bvs_fit <- function(data, Xmat, sensitivity, specificity,
                    b, om1, om2, niter, psample, initsurv,
                    nreport = 10000) {
  if (niter < 10000) stop("Too small niter (at least 10000)")
  Dm <- icensmis:::dmat(data$ID, data$time, data$result,
                        sensitivity, specificity, 1)
  fit <- icensmis:::bayesmc(Dm, Xmat, b, om1, om2, niter,
                            psample, initsurv, nreport, icensmis:::fitsurv)
  icensmis:::gamma_mean(fit, 20000)
}

#' Function to obtain BVS fitting of the data assuming perfect test
#' Note it only works for NTFP, likelihood function invalid for MCAR
#' @return a vector of mean gamma for each feature
#'
bvs_fit_perfect <- function(data, Xmat, sensitivity, specificity,
                            b, om1, om2, niter, psample, initsurv,
                            nreport = 10000) {
  if (niter < 10000) stop("Too small niter (at least 10000)")
```

```
  Dm <- icensmis:::dmat(data$ID, data$time, data$result,
                        1, 1, 1)
  fit <- icensmis:::bayesmc(Dm, Xmat, b, om1, om2, niter,
                            psample, initsurv, nreport, icensmis:::fitsurv)
  icensmis:::gamma_mean(fit, 20000)
}

#' Function to produce result for one simulation with different methods
#' @param cuminc cumulative incidence rate
#' @param sensitivity sensitivity
#' @param specificity specificity
#'
sim <- function(cuminc, sensitivity, specificity) {
  # randomly select features from features
  nbeta <- ncol(Xmat)
  nbiomarker <- length(beta_value)
  ids <- sample(nbeta, nbiomarker)
  betas <- rep(0, nbeta)
  betas[ids] <- beta_value

  # generate simulation data: NTFP for all cases
  data <- simdata(Xmat, 1 - cuminc, testtimes,
                  sensitivity, specificity, betas,
                  design = "NTFP")

  # Cox fit
  cox_result <- cox_fit(data, Xmat)
  cox_ranks <- rank(-abs(cox_result), ties.method = "random")
  cox_result <- data.frame(method = "Cox", ids = ids,
                           ranks = cox_ranks[ids],
                           stringsAsFactors = FALSE)

  # Univariate fit
  unv_result <- unv_fit(data, Xmat)
  names(unv_result) <- NULL
  unv_ranks <- rank(-unv_result, ties.method = "random")
  unv_result <- data.frame(method = "UNV", ids = ids,
                           ranks = unv_ranks[ids],
                           stringsAsFactors = FALSE)

  # RSF fit
  rsf_result <- rsf_fit(data, Xmat)
  rsf_ranks <- rank(-rsf_result, ties.method = "random")
  rsf_result <- data.frame(method = "RSF", ids = ids,
                           ranks = rsf_ranks[ids],
                           stringsAsFactors = FALSE)
```

```
  # BVS fit
  # initsurv <- (1-cuminc)^exp(.35*5)
  initsurv <- 1 - cuminc
  bvs_result <- bvs_fit(data, Xmat, sensitivity, specificity,
                        b, om1, om2, niter, psample, initsurv)
  bvs_ranks <- rank(-bvs_result, ties.method = "random")
  bvs_result <- data.frame(method = "BVS", ids = ids,
                           ranks = bvs_ranks[ids],
                           stringsAsFactors = FALSE)

  # BVS fit assuming perfect
  bvs_result_perfect <- bvs_fit_perfect(data, Xmat, sensitivity, specificity,
                                        b, om1, om2, niter, psample, initsurv)
  bvs_ranks_perfect <- rank(-bvs_result_perfect, ties.method = "random")
  bvs_result_perfect <- data.frame(method = "BVS_PERFECT", ids = ids,
                                   ranks = bvs_ranks_perfect[ids],
                                   stringsAsFactors = FALSE)

  # OUTPUT
  result <- rbind(cox_result, unv_result, rsf_result,
                  bvs_result, bvs_result_perfect)
  result_ntfp <- data.frame(cuminc = cuminc,
                            sensitivity = sensitivity,
                            specificity = specificity,
                            design = "NTFP",
                            result)

  # MCAR only for BVS
  data_mcar <- simdata(Xmat, 1 - cuminc, testtimes,
                       sensitivity, specificity, betas,
                       design = "MCAR")
  bvs_result_mcar <- bvs_fit(data_mcar, Xmat, sensitivity, specificity,
                             b, om1, om2, niter, psample, initsurv)
  bvs_ranks_mcar <- rank(-bvs_result_mcar, ties.method = "random")
  bvs_result_mcar <- data.frame(method = "BVS", ids = ids,
                                ranks = bvs_ranks_mcar[ids],
                                stringsAsFactors = FALSE)
  result_mcar <- data.frame(cuminc = cuminc,
                            sensitivity = sensitivity,
                            specificity = specificity,
                            design = "MCAR",
                            bvs_result_mcar)
  rbind(result_ntfp, result_mcar)
}
```

config.yaml:

```
Xmat_File: sim_data/Xmat.rda

beta_value: [1, 1, 1, 1, 1]
testtimes: [1, 2, 3, 4]

b: 1
om1: 5
om2: 100
niter: 100000
psample: 0.3

params: [[0.1, 0.75, 1.00],
         [0.1, 0.61, 0.995],
         [0.1, 1.00, 0.90],
         [0.3, 1.00, 1.00],
         [0.3, 0.75, 1.00],
         [0.3, 0.61, 0.995],
         [0.3, 1.00, 0.90],
         [0.1, 1.00, 1.00]]
```

This sim.R code needs to be ran in batch mode so it can be easily deployed in various environments with R installed. The following script will run the code in 8 threads in parallel and it is repeated 125 times resulting 1,000 simulations for each parameter setting. The results are saved in sim_results folder.

```
Rscript sim.R config.yaml sim_results 125 8
```

sim.R:

```
parms <- commandArgs(trailingOnly = TRUE)
source("utils.R")

# yaml file
load_params(parms[1])

# destination folder to store output result
dest <- parms[2]

# number of reptition
nrep <- as.numeric(parms[3])
if (is.na(nrep)) nrep <- 1

# number of cores
ncore <- as.numeric(parms[4])

# iterate all parameter sets one time
load(Xmat_File)
```

```
onesim <- function() {
  simresult <- lapply(params, function(x) {
    print(sprintf("Simulating %s, %s, %s", x[1], x[2], x[3]))
    sim(x[1], x[2], x[3])
  })
  do.call("rbind", simresult)
}

# iterate all parameters sets multiple times
multisim <- function() {
  require(parallel)
  print(sprintf("Job started %s", Sys.time()))
  if (is.na(ncore)) {
    result <- lapply(1:1, function(i)
      onesim())
  } else {
    result <- mclapply(1:ncore, function(i)
      onesim(), mc.cores = ncore)
  }
  result <- do.call("rbind", result)
  print(sprintf("Job finished %s", Sys.time()))
  # output
  seq <- as.character(Sys.time())
  seq <- gsub("[[:punct:]]", "_", seq)
  if (!file.exists(dest)) dir.create(dest)
  filename <- sprintf("%s/result%s.rda", dest, seq)
  save(result, file = filename)
}

for (i in 1:nrep) {
  multisim()
}
```

5 Application: WHI Clinical Trial and Observational Study SHARe

Within each race/ethnicity subgroup, the following two step procedure was followed to reduce dimensionality: First, SNPs that satisfied one or more of the following three criteria were excluded from the analysis: (i) more than 1% missing values; or (ii) less than 5% minor allele frequency; or (iii) a Hardy-Weinberg equilibrium test p value less than 10^{-6}. Second, the univariate association of each SNP with incident diabetes was estimated using a two-sided likelihood ratio test based on the observed data likelihood in equation (3) implemented in the R package *icensmis* [1]. The SNPs were ranked by increasing p value and the top 300,000 SNPs were included in our analysis. All missing genotypes were imputed to be the homozygous wildtype ("AA"). Each SNP was coded as a numeric variable by setting genotype "AA" as 0, genotype "Aa" as 1, and "aa" as 2.

To quantify the extent of genetic variability within each self-reported race category (African American and Hispanic American), we carried out a principal components analysis and extracted the top two principal components [2]. In the African American dataset, the first and second principal components (PC1, PC2) accounted for 1.9% and 0.1% of the total variability, respectively. Similarly in the Hispanic American dataset, the first and second principal components (PC1, PC2) accounted for 2.1% and 0.9% of the total variability, respectively. Figures 2-3 in the Supplement show the scatterplot of PC1 versus PC2, estimated separately from the 300K SNPs in the African American and Hispanic American datasets within the WHI. The top two principal components were incorporated into the analysis to account for population stratification within each self-reported race/ethnicity group.

The 300K SNPs included in each analysis were ranked from most to least important by the following three methods:

1 Proposed BVS algorithm (BVS_e), where $\varphi_1 = 0.61, \varphi_0 = 0.995$. The values of the hyper-parameters governing the prior distributions were set to the following: $b = 1.0, w_1 = 5, w_2 = 100$. We note that the choice of $b = 1$ allows the log hazard ratios to vary between -2 and 2 with probability 0.95. SNPs were ranked based on the posterior probability of inclusion.

2 BVS algorithm ($\mathrm{BVS}_{perfect}$) assuming perfect self-reports, where $\varphi_1 = 1.0, \varphi_0 = 1.0$. The values of the hyper-parameters governing the prior distributions were set to the following: $b = 1.0, w_1 = 5, w_2 = 100$. We note that the choice of $b = 1$ allows the log hazard ratios to vary between -2 and 2 with probability 0.95. SNPs were ranked based on the posterior probability of inclusion.

3 Univariate (SNP by SNP) analysis based on the likelihood in Equation (3) and implemented in the R package *icensmis* [1] (denoted *icensmis*). In this analysis, we set $\varphi_1 = 0.61, \varphi_0 = 0.995$. The first two principal components were included as additional covariates. SNPs were ranked based on a two-sided likelihood ratio test p value.

4 Univariate analysis (SNP by SNP) analysis based on a Cox PH model. The time origin for each participant was set to the time of entry into the WHI. The time-to-event outcome was defined as the minimum of the time to the first positive self-report (observed event) and the time to the last observation (censored event). The first two principal components were included as additional covariates. SNPs were ranked based on a two-sided likelihood ratio test p value.

The computing time for the MCMC analysis of the dataset of African American WHI participants (300K SNPs on 6704 participants) on a 2013 Mac Pro 3.5 GHz 6-Core Intel Xeon E5 computer was about 10 days. The computing time for the dataset of Hispanic American WHI participants (300K SNPs on 3169 participants)

on the same computer was about 5 days. In each analysis, the MCMC algorithm was run up to 5 million iterations and the first 1 million iterations were discarded as burn-in. The MCMC algorithm was run using R [3].

Author details
[1]Department of Biostatistics and Epidemiology, University of Massachusetts, Amherst, MA, USA. [2]Department of Mathematics and Statistics, Georgetown University, Washington, DC, USA. [3]Biostatistics Center, Division of Clinical Research, Massachusetts General Hospital Research Institute, Boston, MA, USA. [4]Department of Medicine, University of Massachusetts Medical School, Worcester, MA, USA.

References

1. Gu X and Balasubramanian R. icensmis: Study design and data analysis in the presence of error-prone diagnostic tests and self-reported outcomes r package version 1.1. *R* 2013; .
2. Price AL, Patterson NJ, Plenge RM et al. Principal components analysis corrects for stratification in genome-wide association studies. *Nature Genetics* 2006; 38(8): 904–909.
3. R Core Team. *R: A Language and Environment for Statistical Computing*. R Foundation for Statistical Computing, Vienna, Austria, 2013. `http://www.R-project.org/`.

Table 1 Convergence diagnostics based on the Heidelberg and Welch test for the sampled values of ω. The number of total iterations and number to be discarded as burn-in estimated using the Raftery and Lewis's diagnostic test.

Cumulative incidence	Sensitivity	Specificity	p value*	Burn in	Number of iterations
0.1	1.00	1.00	0.19	88	110858
0.1	0.75	1.00	0.63	56	70616
0.1	0.61	0.995	0.07	64	89536
0.1	1.00	0.90	0.26	33	43879
0.3	1.00	1.00	0.14	124	160363
0.3	0.75	1.00	0.05	39	53430
0.3	0.61	0.995	0.63	42	59402
0.3	1.00	0.90	0.08	92	123671

*: p value from the Heidelberg and Welch test [3]

Table 2 Probability of ranking among the top five metabolites by posterior probability of inclusion, for metabolites that are associated with outcome. CIR denotes the cumulative incidence rate in the reference group, RSF denotes Random Survival Forests, BVS denotes the proposed BVS algorithm assuming perfect self-reports and BVS$_e$ denotes the proposed BVS procedure. NTFP denotes a study design setting in which all self-reports following the first positive result are discarded and NMISS denotes the setting where there are no missed visits.

CIR	sensitivity (φ_1)	specificity (φ_0)	RSF	BVS	BVS$_e$ NTFP	BVS$_e$ NMISS
0.1	1	1	$0.46(\pm0.01)$	$0.76(\pm0.01)$	$0.76(\pm0.01)$	$0.76(\pm0.01)$
0.1	1	0.9	$0.34(\pm0.01)$	$0.38(\pm0.01)$	$0.42(\pm0.01)$	$0.72(\pm0.01)$
0.1	0.75	1	$0.45(\pm0.01)$	$0.69(\pm0.01)$	$0.73(\pm0.01)$	$0.74(\pm0.01)$
0.1	0.61	0.995	$0.44(\pm0.01)$	$0.60(\pm0.01)$	$0.65(\pm0.01)$	$0.68(\pm0.01)$
0.3	1	1	$0.53(\pm0.01)$	$0.87(\pm0.01)$	$0.87(\pm0.01)$	$0.87(\pm0.01)$
0.3	1	0.9	$0.44(\pm0.01)$	$0.57(\pm0.01)$	$0.66(\pm0.01)$	$0.84(\pm0.01)$
0.3	0.75	1	$0.51(\pm0.01)$	$0.76(\pm0.01)$	$0.83(\pm0.01)$	$0.84(\pm0.01)$
0.3	0.61	0.995	$0.50(\pm0.01)$	$0.69(\pm0.01)$	$0.78(\pm0.01)$	$0.78(\pm0.01)$

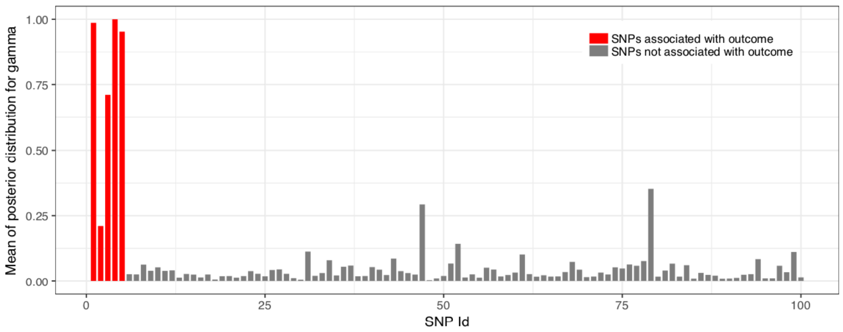

Figure 1 Barplot of the posterior probability of inclusion for each of 100 SNPs, where $1 - S_{J+1} = 0.30, \varphi_1 = 0.61, \varphi_0 = 0.995$ and there are no missed visits. The results shown here are for a $representative\ simulation$ as the identity of SNPs with true associations ($\beta = 0.7$) varies between simulations. 'Red' indicates true associations and'grey' indicates SNPs not associated with outcome.

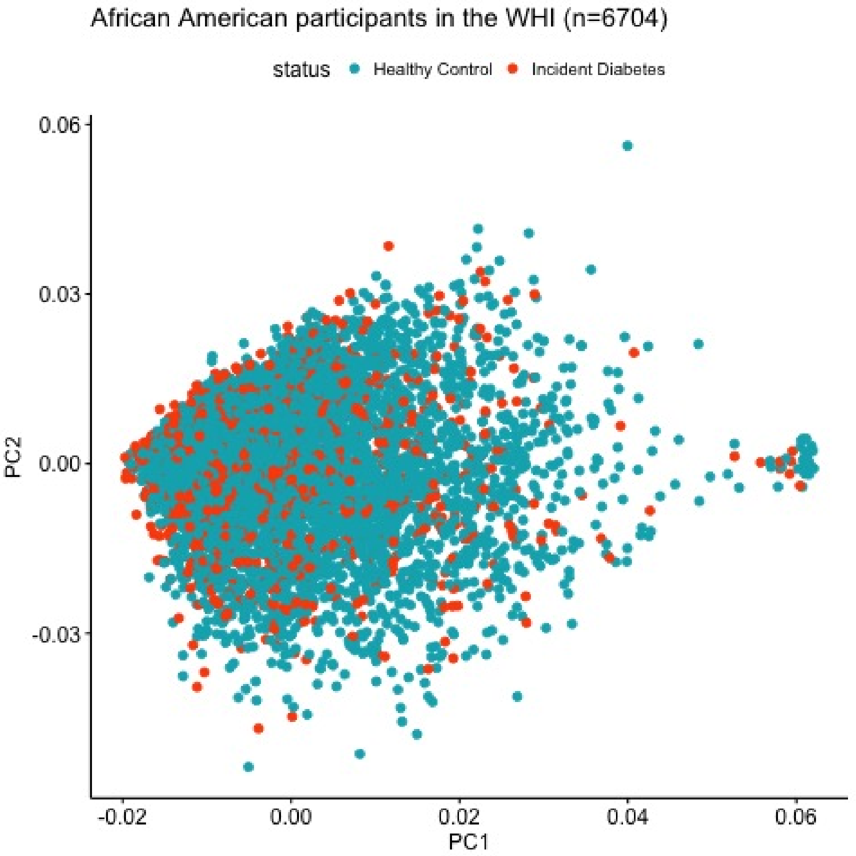

Figure 2 WHI Clinical Trial and Observational Study SHARe: Scatter plot of the first two principal components (PC1, PC2) based on 300,000 SNPs in 6704 African American women in the WHI.

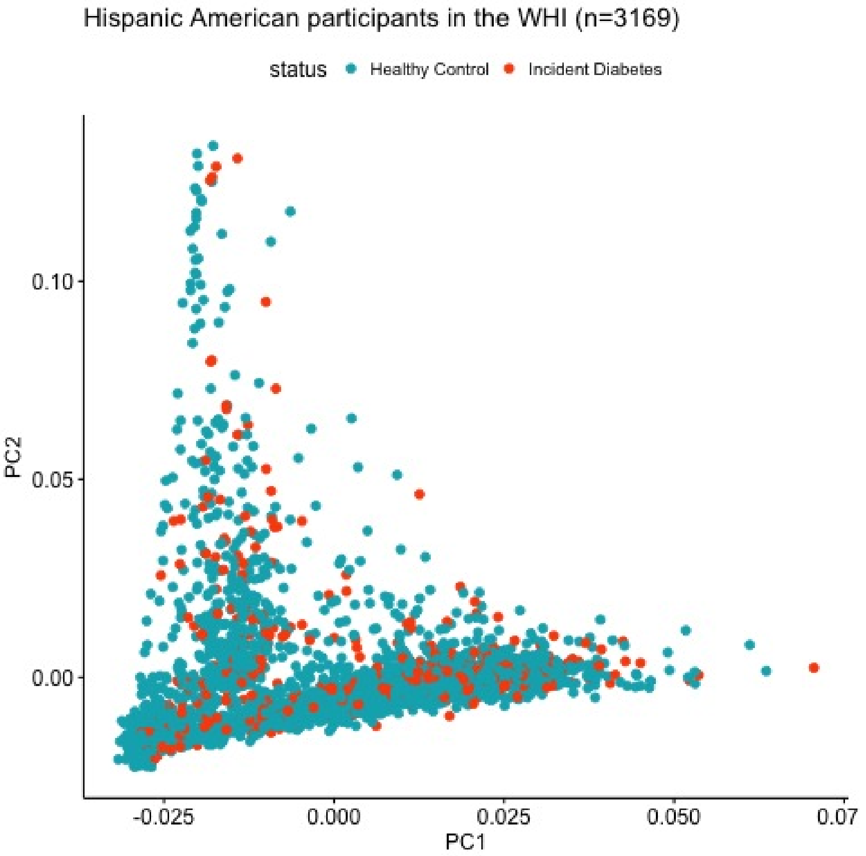

Figure 3 WHI Clinical Trial and Observational Study SHARe: Scatter plot of the first two principal components (PC1, PC2) based on 300,000 SNPs in 3169 Hispanic American women in the WHI.

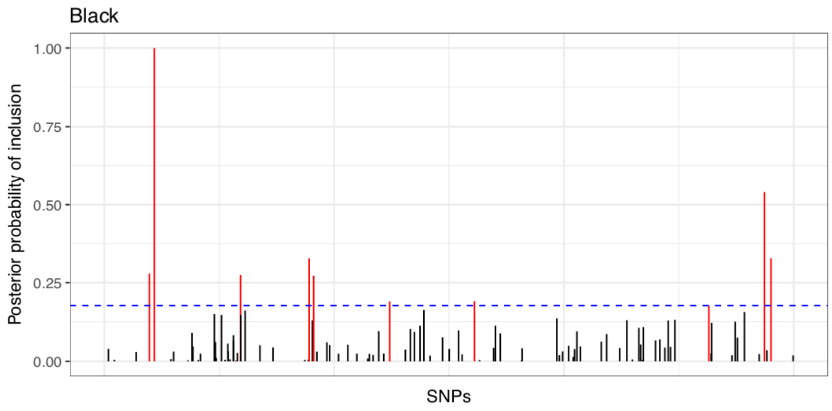

Figure 4 WHI Clinical Trial and Observational Study SHARe: Bar plot of the posterior probability of inclusion for each of the 300K SNPs included in the BVS$_e$ analysis, in the dataset of African American women ($n = 6704$) in the WHI. The horizontal line denotes the threshold separating the top 10 SNPs from the rest.

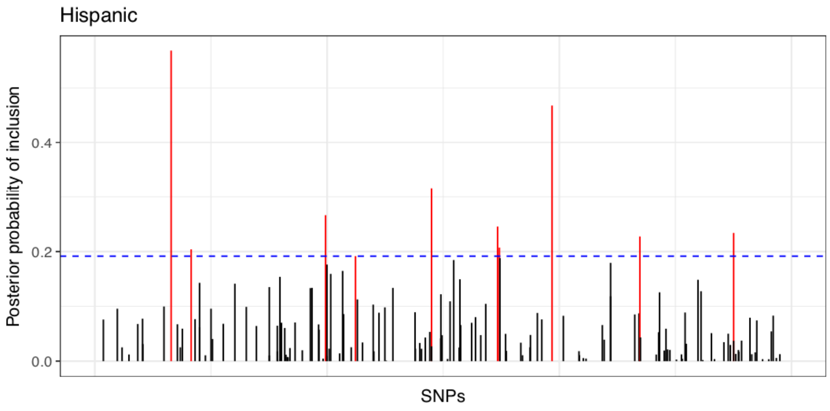

Figure 5 WHI Clinical Trial and Observational Study SHARe: Bar plot of the posterior probability of inclusion for each of the 300K SNPs included in the BVS$_e$ analysis, in the dataset of Hispanic American women ($n = 3169$) in the WHI. The horizontal line denotes the threshold separating the top 10 SNPs from the rest.

References

1. Gu X, Ma Y and Balasubramanian R. Semi-parametric time to event models in the presence of error-prone, self-reported outcomes - with application to the women's health initiative. *Annals of Applied Statistics* 2015; 9(2): 714 – 730.
2. Guo Y and Balasubramanian R. Comparative evaluation of classifiers in the presence of statistical interaction between features in high-dimensionality data settings. *International Journal of Biostatistics* 2012; 8.
3. Heidelberg P and Welch PD. Simulation run length control in the presence of an initial transient. *Operations Research* 1983; 31, 1109-1144.
4. Raftery, AE and Lewis, SM One long run with diagnostics: Implementation strategies for Markov chain Monte Carlo. *Statistical Science* 1992; 7, 493-497.
